# A Numerical Investigation on the Influence of Film-Cooling Hole Inclination Angle on the Stress Field of Surrounding Thermal Barrier Coating

**DOI:** 10.3390/ma18174079

**Published:** 2025-08-31

**Authors:** Zhengyu Shi, Yuhao Jia, Xing He, Zegang Tian, Yongbao Liu

**Affiliations:** College of Power Engineering, Naval University of Engineering, Wuhan 430033, China; bro_ten@163.com (Z.S.); hexing_mail@163.com (X.H.); 18835219305@139.com (Z.T.)

**Keywords:** thermal barrier coating (TBC), film cooling, numerical simulation, creep, stress analysis

## Abstract

Thermal barrier coating (TBC) around film-cooling holes is a key failure location for turbine blade TBC. This study built a numerical model. The model used conjugate heat transfer (CHT) and sequential thermal-stress calculation methods. It analyzed the temperature and stress fields in the TBC around film-cooling holes. The holes had different inclination angles (30°, 45°, and 60°). It also explored the balance between cooling effectiveness and stress at these angles. Results show that increasing the film-cooling hole angle reduces the cooling film coverage area significantly. Cooling effectiveness becomes worse. The temperature field near the holes is complex. Sharp temperature gradients exist there. An inverse temperature gradient appeared in the top coat (TC) layer at the hole exit. Stress in the TBC was analyzed next. Analysis was conducted under rated operating conditions. Analysis was also completed after 500 h of creep under these conditions. Stress concentration around the holes is obvious. At room temperature, Mode I cracks easily form upstream of the holes. Mode II cracks easily form downstream. Under rated conditions, mixed-mode cracks (I + II) easily form downstream. The coating experiences larger stress at room temperature. This means that the coating is more likely to spall during cooling. Increasing the hole angle can reduce stress concentration. It can also lower the chance of crack formation. However, a larger angle increases the normal momentum of the cooling jet. This reduces film coverage. Therefore, after considering both cooling effectiveness and TBC failure, the 45° film-cooling hole is optimal.

## 1. Introduction

A gas turbine is a power device that converts internal energy of fuel into mechanical energy. It has advantages such as high power density and fast start-up speed. Gas turbines are widely used in aerospace propulsion, marine propulsion, and distributed power generation. The turbine is the component responsible for performing work through gas expansion. The turbine guide vanes directly endure the impact of high-temperature gases from the combustion chamber outlet [1]. Increasing the turbine inlet temperature effectively enhances gas turbine power and thermal efficiency. However, excessively high operating temperatures pose significant challenges to the reliability of hot-section components like combustors and turbine blades. Current high-temperature materials are approaching their performance limits [2]. Therefore, implementing thermal protection for gas turbines is essential. Thermal barrier coating (TBC) and film cooling are both highly effective thermal protection technologies [3,4]. The TBC system is composed of the alloy substrate (SUB), the ceramic top coat (TC), the bond coat (BC), and the thermally grown oxide (TGO) layer that forms between the TC and the BC. This coating system can reduce the temperature of the metal substrate by approximately 300 K. The thermal insulation provided by TBC allows the gas inlet temperature to significantly exceed the melting point of the metal blades. This capability thereby improves overall engine performance.

The stress failure and spallation of TBC significantly affect the service life of turbine blades. Factors such as thermal expansion mismatch, thermal cycling, and high-temperature oxidation [5,6,7] can lead to TBC failure. Many researchers have conducted extensive studies on the failure mechanisms of TBC. Tsui [8] established a model relating to the curvature of the plate and the residual stress during the step-by-step deposition process of TBC based on the linear elastic theory of beam force and moment balance. Zhou and Hashida [9] developed a two-dimensional plane model based on a hollow cylinder, which considers the effects of temperature gradient, TGO thickening, material creep, and plastic deformation on coating stress. Later, Mao et al. [10] further developed a two-dimensional stress calculation model that considers the effects of temperature gradient, TGO thickening, material creep, plastic deformation, as well as the influence of moment and curvature on residual stress. Zhang et al. [11] used a concentric circle model to theoretically study the impact of oxide layer thickness on interface stress. Hsueh [12,13] introduced coating thickness and established a residual-stress calculation model for multilayer structures formed by thermal mismatch, which calculates average residual stress through changes in beam curvature. Yu [14] developed a finite element model for air plasma sprayed (APS) TBC to study the residual-stress level and distribution at the TC–TGO interface and BC–TGO section under thermal cycling conditions. Balint and Hutchinson [15] focused on the non-uniform growth of the oxide layer and established an analytical model for coating stress by considering factors such as creep, thermal mismatch, and phase transformation. Rösler and Bäker [16,17] studied the impact of changes in material parameters related to creep and plastic deformation models on the stress distribution of TBC, showing that creep and plastic deformation can relax coating stress. Other researchers have studied the effects of interface morphology, material properties, TGO thickening, and other factors on coating stress based on mesoscopic models [18,19,20,21,22,23].

The above studies focus on the performance of individual TBC, yielding many valuable insights. However, actual turbine blades use a combination of film cooling and TBC protection technologies. Therefore, studying the coating performance alone is insufficient; it is necessary to analyze the thermodynamic effects of film-cooling holes in depth. Additionally, many studies on film cooling simplify the effects of heat conduction and internal convection, treating the wall surface as an adiabatic boundary condition. In reality, turbine components are not adiabatic, and the adiabatic wall treatment does not consider the conjugate coupling effects of external film cooling, internal convection, and wall heat conduction in real environments. Conjugate calculations can account for the mutual influence of heat transfer and fluid flow, making the use of conjugate heat transfer methods more accurate in assessing film cooling performance and its impact on surrounding TBC. For TBC with film cooling, Bohn et al. [24] used conjugate heat transfer (CHT) methods and adiabatic methods to simulate fluid flow over flat plates with TBC and film cooling. Under any conditions, compared to the impact of reduced surface area, the thermal conductivity of the solid and the cooling driving temperature are the main factors affecting cooling effectiveness. Mensch et al. [25,26] studied the cooling performance of blade end walls under different blowing ratios and with or without TBC using a coupled heat transfer model. The study included internal impingement cooling and external film cooling, and the experimental and computational results showed that TBC has a significant effect, reducing wall temperature in all cases. Adding TBC greatly improves overall cooling effectiveness and makes internal cooling more efficient.

Stress and failure of TBC around film-cooling holes are also key research areas. Kim et al. [27] and Lee et al. [28] used numerical methods. They studied how material properties affect temperature and thermal stress in TBC near holes. They combined locally measured heat transfer coefficients from experiments. They analyzed TBC spallation near vertical film-cooling holes. Based on experimental data, they developed a correlation to predict peeling stress. Jiang et al. [29] used CHT calculations. They obtained the temperature field in a TBC film-cooling system under operating conditions. They built a finite element model (FEM). This model calculated thermal stress in the TBC at high temperatures and after 500 h of creep. They found interfacial peeling stress and shear stress at the hole edges. These stresses can initiate Mode I and Mode II edge cracks during heating or cooling cycles. Qiao et al. [30] performed a numerical study. They studied the thermomechanical behavior of a TBC film-cooling system under service conditions. They used CHT to obtain the TBC temperature field distribution. They combined this with material properties: TGO growth, plasticity, and creep. They analyzed stress in TBC at film-cooling hole edges under real operating conditions. Dai et al. [31] established a CHT numerical model. This model determined the temperature distribution in TBC with film-cooling systems. They used finite element analysis (FEA). They studied the infiltration behavior of calcium-magnesium-alumino-silicate (CMAS). They discussed CMAS penetration effects. These effects include impacts on temperature distribution, TGO growth rate, and residual stress. Meng et al. [32] considered a non-uniform temperature field. They built an analytical model. This model predicts residual stress in TBC film-cooling systems. Liu et al. [33] used a thermal–fluid–structure coupling method. They systematically studied cooling effectiveness and interfacial stress in TBC film-cooling systems under different curvatures. Studies covered both steady-state and transient conditions. The durability of TBC with film-cooling holes depends on TBC insulation and hole geometry. Several factors cause TBC failure near holes [32,34]. These include severe temperature gradients causing complex 3D stress, free-edge effects from hole geometry, and thermal mismatch from different layer properties. Many studies exist on how hole inclination angle affects film-cooling effectiveness. Fewer studies examine its effect on TBC. Therefore, further research is needed. This research should investigate the thermomechanical behavior of TBC around film-cooling holes at different inclination angles. This is essential to improving turbine blade service life.

This study uses conjugate heat transfer (CHT) and sequential thermal-stress calculation methods to conduct a numerical investigation of a TBC flat plate model with film-cooling holes. It analyzes the temperature and stress fields of the TBC around film-cooling holes at different angles (30°, 45°, and 60°). Under rated conditions, this study examines the temperature distribution of TBC at various angles. It also considers the plasticity and creep characteristics of the material, comparing the residual stress of TBC with different film-cooling hole angles after 500 h of creep under rated conditions. This study analyzes the interface stress of each TBC layer and identifies potential crack locations and forms. Finally, it explores the balance between cooling effectiveness and stress for film-cooling holes at different inclination angles.

## 2. Numerical Model

### 2.1. Geometric Model

**Figure 1:**
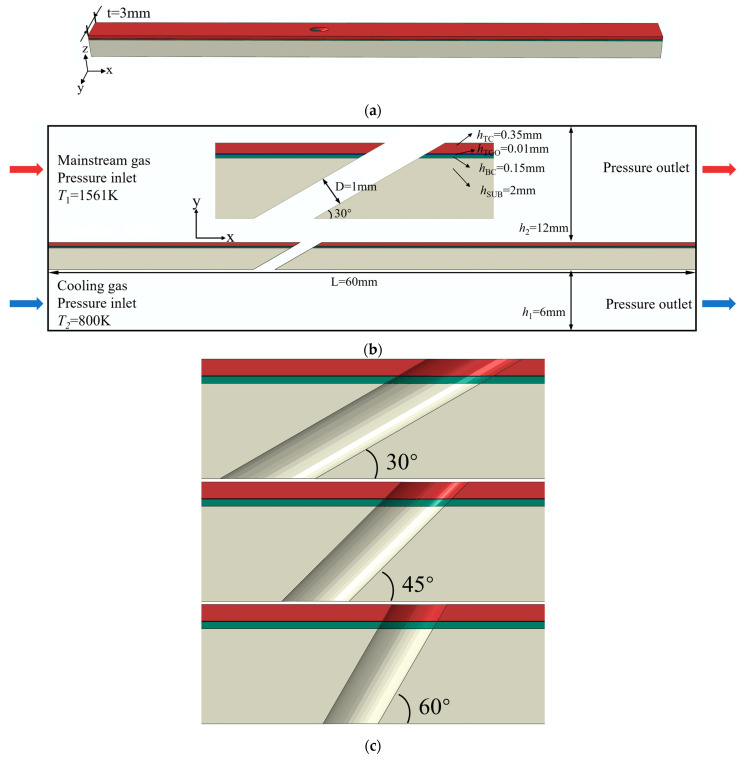
Geometry and boundary conditions of the model. (**a**), Schematic of the flat plate model (**b**), Geometry and boundary conditions (30° hole case) (**c**), Film-cooling hole models with 30°, 45°, and 60° inclination angles.

Figure 1 shows the geometry and boundary conditions of the model. A TBC flat plate model with film-cooling holes is selected as the research subject. Thermal barrier coating uses a typical APS-YZS coating, consisting of a 0.35 mm ceramic layer (TC), a 0.01 mm oxide layer (TGO), a 0.15 mm bond coat (BC), and a 2 mm high-temperature alloy substrate (SUB). The plate is 60 mm long and 3 mm wide, with cylindrical film-cooling holes of diameter D = 1 mm. Models with film-cooling holes at angles of 30°, 45°, and 60° are selected.

### 2.2. CFD Model

Thermal barrier coatings with film-cooling holes involve three heat transfer processes in practical applications: thermal convection between hot/cold gases and the TBC, thermal conduction between the layers of the TBC, and thermal radiation. It is necessary to use fluid–solid coupled heat transfer methods to simultaneously calculate the temperature fields in both the fluid and solid domains. In CFD modeling, the conjugate heat transfer (CHT) method is employed to handle the interactions between the fluid and the solid. During the conjugate process, the interface between the fluid and the solid satisfies:

(1) $$T_{f}=T_{S}$$

(2) $$-k_{s}\frac{\partial T_{S}}{\partial n}=k_{f}\frac{\partial T_{f}}{\partial n}$$

where *T_f_* and *T_S_* represent the temperatures at the interface in the fluid domain and solid domain, respectively; *k_s_* and *k_f_* denote the thermal conductivities of the solid and fluid domains, respectively; and ***n*** indicates the normal direction of the interface.

Due to the high temperature of the combustion gases, thermal radiation must be considered in CFD calculations. The discrete ordinates method (DO radiation model) is used for radiation calculations, and the gas absorption coefficient is determined using the weighted sum of gray gases model (WSGGM).

In film cooling, to quantify the flow relationship between the cooling gas and the mainstream, the blowing ratio *M* of the cooling jet to the mainstream is defined as:

(3) $$M=\frac{\rho_{c}u_{c}}{\rho_{\infty }u_{\infty }}$$

where $\rho_{c}$ and $u_{c}$ are the density and velocity of the cooling jet, while $\rho_{\infty }$ and $u_{\infty }$ are the density and velocity of the mainstream.

CFD calculations are performed using Fluent 2021R1 software, with the shear stress transport (SST) model selected for turbulence modeling. To closely simulate real working conditions, the rated operating conditions of the turbine blade are chosen as boundary conditions. The height of the mainstream channel is 12 mm, and the height of the cooling flow channel is 6 mm, with both having a width of 3 mm. The turbulence intensity at the inlet of the mainstream and cooling gas is set to 10%. The mainstream is set as a pressure inlet with a total pressure of 1.84 MPa, and the outlet is a pressure outlet with a total pressure of 1.80 MPa and a total temperature of 1561 K. The cooling flow outlet pressure is set to 1.80 MPa, and the inlet total pressure is adjusted continuously to ensure that the blowing ratio *M* remains constant at 1 under any inclination angle, with a total temperature of 800 K. The sides of the flow channels are set as translational periodic symmetry conditions. The interface between the fluid domain and solid domain is set for conjugate heat transfer, while other surfaces are set as adiabatic walls.

**Figure 2:**
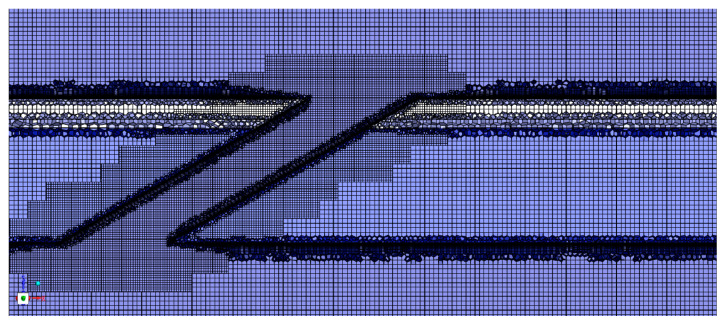
CFD model mesh (30° hole case).

Figure 2 illustrates the mesh structure near the film hole, utilizing a poly-hexcore mesh division method. This mesh division allows for the connection of polyhedral and hexahedral meshes at common nodes, eliminating interfaces with non-common nodes. It enhances the efficiency and accuracy of the solution while reducing the number of meshes. The mesh division at common nodes is applied between the fluid domain and the solid domain. Boundary layers are added within the film hole and at the fluid–solid interface wall. The first layer of the boundary layer has a thickness of 0.001 mm, with a growth rate of 1.2, and the number of boundary layers is 8 and 20, respectively, to ensure the y+ value near the wall is less than 1. Additionally, the material parameters for each layer of the solid domain’s TBC are shown in Table 1.

### 2.3. FE Model

**Figure 3:**
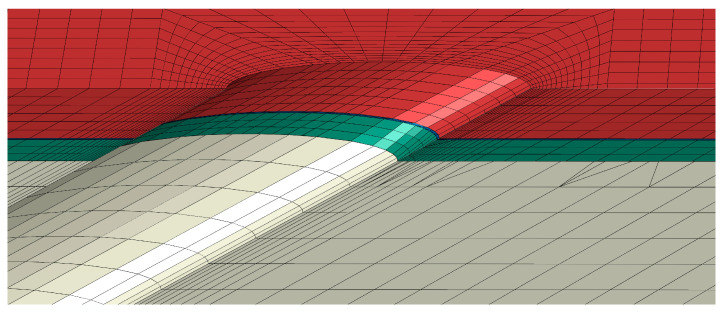
FE model mesh (30° hole case).

After calculating the temperature field of the solid domain through CFD, the temperature field is interpolated into Abaqus 6.14 software as the thermal initial condition for thermomechanical calculations. The solid domain uses three-dimensional eight-node linear hexahedral elements (C3D8) mesh, as shown in Figure 3. To reduce computational load, local mesh refinement is applied near the film hole, with the TGO layer using two layers of mesh, and the total number of mesh elements is 100,000. Each material layer is set as isotropic, with material properties as shown in Table 1. The TC and TGO layers are primarily composed of oxide ceramics, which exhibit almost no plasticity, while the BC and SUB layers are alloy materials capable of plastic deformation, with plasticity parameters as shown in Table 2. Plasticity changes significantly with temperature, with yield stress decreasing sharply at high temperatures, increasing material plasticity. Creep refers to the phenomenon where a material undergoes progressive, irreversible plastic deformation over time under high temperatures and constant stress (which can be below the yield strength). TBC experiences creep deformation at high temperatures, which can relieve internal stress to some extent, thus affecting the overall stress distribution. The Norton creep model can be used to describe the creep behavior of materials within the TBC, expressed as:

(4) $${\dot{\varepsilon }}_{cr}=B\cdot \sigma^{m}$$

where ${\dot{\varepsilon }}_{cr}$ and σ represent the creep strain rate and stress, respectively. *B* is the creep coefficient, and *m* is the creep exponent. Table 3 lists the creep parameters of the material as a function of temperature.

### 2.4. Experimental Validation

The model underwent mesh independence verification, resulting in a final total of 3.4 million mesh elements. This study validated the numerical calculation method by comparing it with WANG’s high-temperature experiment [37]. The mainstream gas temperature was 1082.15 K, with a velocity of 15.5 m/s, while the cooling airflow temperature was 689.15 K, with a velocity of 10 m/s. The geometry and boundary conditions of the simulation model were consistent with the experiment, and the mesh scheme and turbulence model were the same as those in the CFD model.

**Figure 4:**
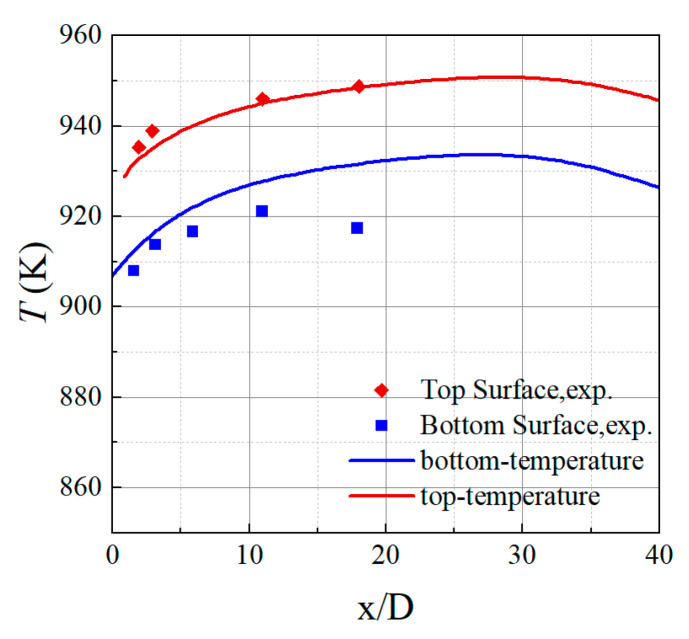
Numerical calculation and experimental validation of film cooling.

Figure 4 shows the temperature distribution along the centerline of the top and bottom surfaces of the plate at the film hole outlet, comparing CFD and experimental results. Considering the effects of radiation, the CFD simulation results showed good consistency with the experimental data, with a maximum deviation of less than 0.5% on the upper surface and less than 1.5% on the lower surface. The agreement between the two reveals the rationality and effectiveness of the CFD model applied in this study.

## 3. Results and Discussion

### 3.1. Flow Characteristics

**Figure 5:**
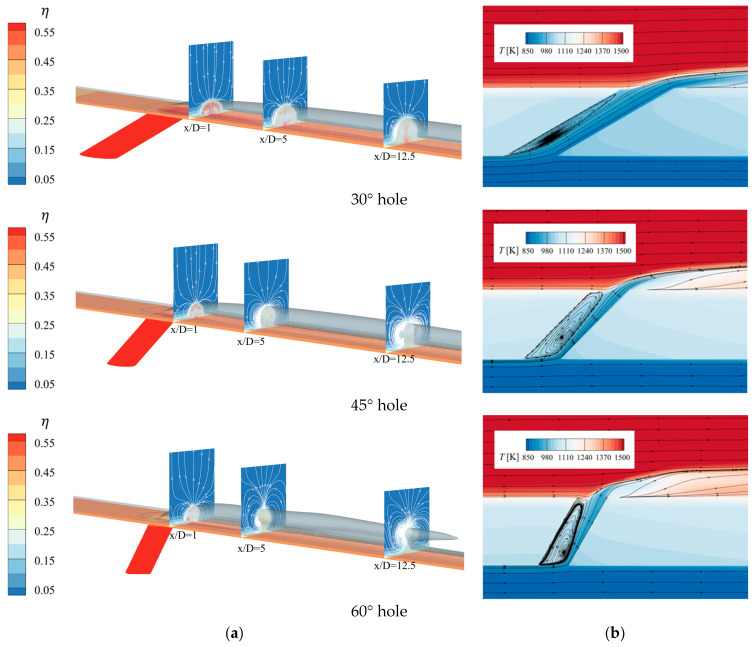
Flow characteristics. (**a**), Flow detail diagram (**b**), The model’s symmetry plane.

Figure 5 shows the main flow details on the wall surface for three cylindrical holes with different inclination angles. This includes the distribution of cooling efficiency η, the η = 0.2 iso-surface, and cross-sections at three different positions along the flow direction x/D. It also includes the temperature field and streamline distribution on the model’s symmetry plane. Here, x is the distance along the flow direction from the film hole exit, and D is the diameter of the film hole. Observing the flow details in Figure 5a, the cooling gas ejected from the film holes mixes with the mainstream, forming a cooling jet that protects the wall surface. The vortices at the x/D cross-section reveal that due to the interaction between the mainstream and the cooling jet, the mixed fluid rotates in opposite directions, forming a kidney-shaped vortex (CVP). As the flow develops downstream, the CVP lifts and separates from the wall, and the distance between the vortices increases, causing the temperature near the wall to rise gradually and the film cooling effectiveness to decrease significantly. At x/D = 12.5 for the 30° hole, the CVP has already separated. With an increase in the inclination angle of the holes, the CVP lifts more significantly along the flow direction, and the distance between them becomes closer. The length of the film in the flow direction shortens, and the spanwise width narrows, causing the cooling film to be lifted significantly, failing to effectively protect the wall surface. The 30° hole exhibits the least CVP effect, with the cooling jet showing high wall adherence and the best spanwise diffusion. This is because, with a constant blowing ratio M, as the inclination angle increases, the streamwise velocity of the cooling gas ejected from the film holes decreases, while the normal velocity increases. After mixing with the mainstream, the streamwise distance shortens, and the lift becomes significant.

Figure 5b presents the temperature field and streamline distribution, showing that the cooling gas primarily affects the flow inside the film holes and near the wall surface. Due to the angle with the cooling flow direction, separation occurs upstream of the film hole entrance, forming a recirculation zone inside, which reduces the actual flow area compared to the film hole cross-section. As the inclination angle increases, the recirculation zone enlarges, further decreasing the actual flow area inside the hole. The wall adherence of the cooling gas near the downstream wall surface of the film hole weakens, leading to an increase in temperature near the wall.

### 3.2. Temperature Distribution

**Figure 6:**
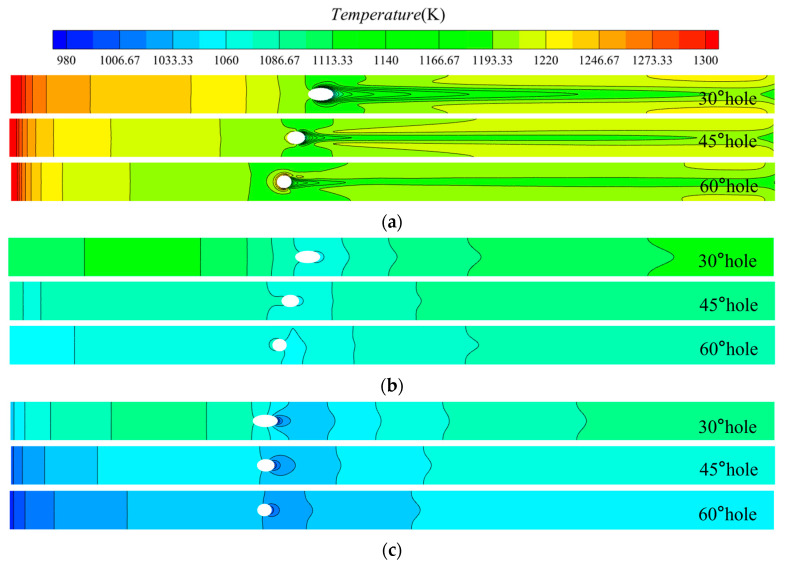

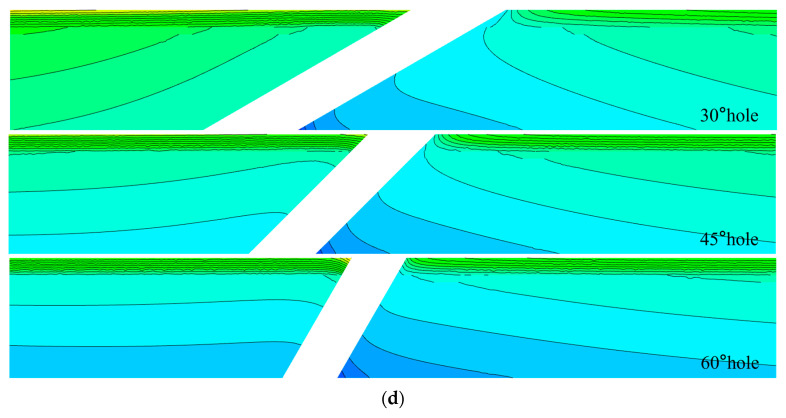
Temperature contour map. (**a**), TC, Top surface (**b**), BC, Bottom surface (**c**), SUB, Bottom surface (**d**), Mid-section around the holes.

Differences in gas flow characteristics affect heat transfer within the solid of thermal barrier coatings. Figure 6 shows the temperature distribution on the surfaces of each layer of thermal barrier coating with film-cooling holes at different inclination angles, as well as the temperature distribution around the mid-section holes. On the TC top surface, the highest temperature appears at the mainstream inlet, with a significant temperature gradient along the mainstream direction. The lowest temperatures are distributed at the downstream exit of the film-cooling holes, where there is a sharp temperature gradient not only along the flow direction but also across the span due to the mixing of cooling jets with the mainstream, resulting in intense thermal convection. A “tongue”-shaped low-temperature region can be observed downstream of the cooling holes, with a temperature difference of up to 120 K between the top and root areas. As the inclination angle of the film-cooling holes increases, the length and width of this region decrease significantly. On the TC top surface, a reverse high-temperature region appears upstream of the 60° film-cooling holes. The temperature drop and gradient on the BC bottom surface and SUB bottom surface are lower than those on the TC top surface, with the lowest temperatures also distributed in the downstream area near the holes. The mid-section around the holes shows a clear temperature gradient along the thickness direction, especially in the TC layer. The three-dimensional temperature distribution near the holes is complex, with very pronounced temperature gradients, and a reverse temperature gradient is observed downstream of the film-cooling holes at the exit. The temperature contour map reveals that the temperature of each layer and the overall temperature of the mid-section decrease as the inclination angle of the film-cooling holes increases. This is because, with a constant blowing ratio M, the total pressure of the cooling flow needs to be increased with the increase in inclination angle, enhancing convective heat transfer between the mainstream and cooling flow, thus showing a decrease in overall temperature in the temperature contour map as the inclination angle increases.

**Figure 7:**
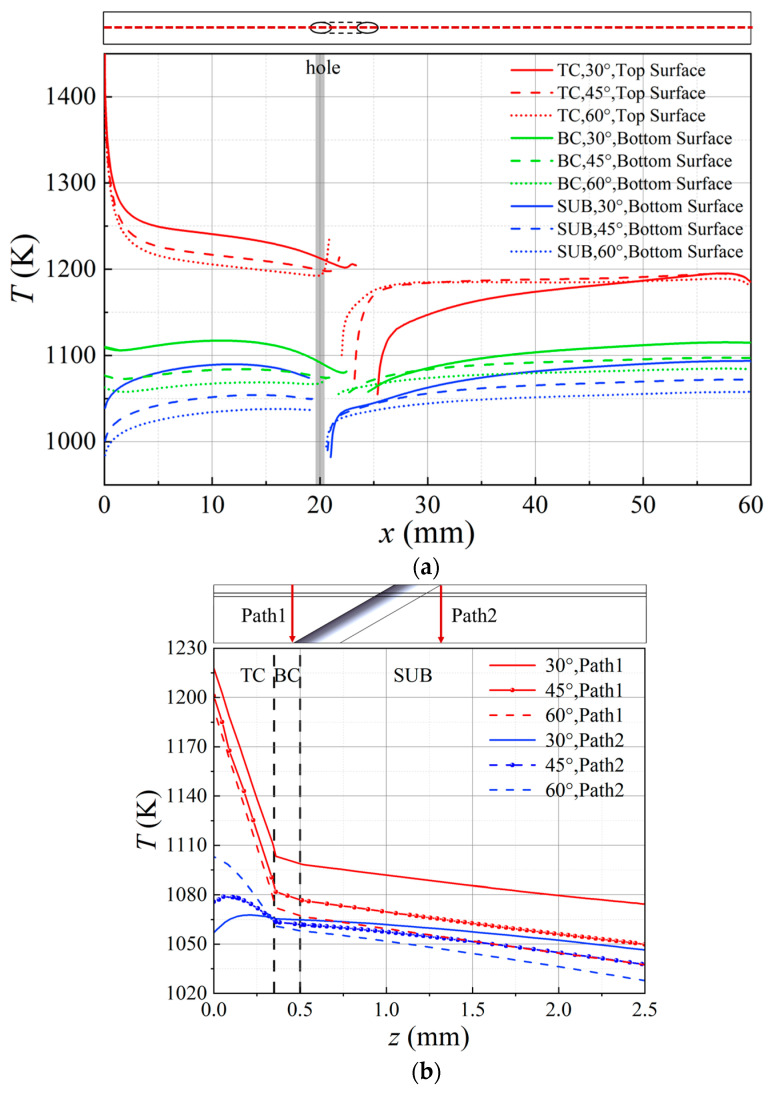
Temperature distribution. (**a**), Along centerline (**b**), Along Path 1 and Path 2.

To further investigate the impact of the inclination angle on the thermal insulation performance of TBC, temperatures along the centerline of each layer were extracted. Temperatures along paths 1 and 2 in the thickness direction were selected, as shown in Figure 7. Figure 7a illustrates the temperature curves along the centerline of each layer’s surface. It is evident that the temperature distribution trends are similar across models with different inclination angles. In the upstream region of the film-cooling holes, without film cooling, the TBC can effectively reduce the metal wall temperature by 150 K, which is the maximum temperature difference between the top surface of the TC layer and the bottom surface of the BC layer. The metal substrate (SUB) has limited thermal insulation capability, with a maximum temperature drop of 50 K between the bottom surface of the BC layer and the bottom surface of the SUB. For the 30° hole, in the downstream region close to the film-cooling hole, the temperature of the TBC’s top surface approaches that of the bottom surface. This occurs because, at the exit of the film-cooling hole, the cooling jet has not yet started to lift, providing good coverage, and is influenced by the cooling gas inside the hole, resulting in an optimal cooling performance. Consequently, the temperature of the TC layer’s top surface is low. The metal substrate (SUB) beneath the coating has a high thermal conductivity. Although the TBC provides thermal insulation protection, other high-temperature areas (downstream of the hole and lateral areas on both sides) can quickly transfer heat, leading to the temperature of the TC layer being close to that of the SUB layer.

Comparing different inclination angles, it can be observed that the overall temperature of each layer decreases as the inclination angle increases. This is due to the differences in convective heat transfer between the cooling flow and the main flow, controlled by the blowing ratio M, under different inclination angles. There are two special regions: near the exit of the film-cooling hole upstream and the downstream region of the film-cooling hole. Near the exit of the film-cooling hole upstream, the 45° and 60° holes exhibit a significant reverse temperature gradient, with the 60° hole showing a larger temperature difference and a more pronounced gradient. In the downstream region of the film-cooling hole, the temperature curve increases with the increase in the inclination angle of the film-cooling hole. This is due to the varying effectiveness of film cooling, with the 30° hole providing the optimal cooling effect, resulting in a more gradual change in the temperature gradient and the longest effective coverage length of the film.

Figure 7b shows the temperature curves along paths 1 and 2. It can be seen that along path 1, the temperature decreases from the TC layer to the SUB layer. The TC layer has good thermal insulation properties, while the BC layer, with material properties similar to metal, has poor insulation. For different film-cooling hole inclination angles, the temperature drop from the TC layer to the SUB layer is the same. Along path 2, the temperatures for the 30° and 45° holes first increase and then decrease, which is different from path 1. A reverse temperature gradient appears in the TC layer along the thickness direction, with the top surface temperature of the 30° hole’s TC layer even lower than the bottom surface. This is because the cold air just exits, resulting in a lower top surface temperature, while the bottom surface is heated by other high-temperature areas, making it warmer than the top surface. Figure 6 and Figure 7 show that there is a very complex three-dimensional temperature distribution near the cooling holes, which can lead to significant local thermal stress. The temperature distribution also varies with different hole inclination angles, further increasing the likelihood of TBC failure.

### 3.3. Thermal Stress at Elevated Temperature

**Figure 8:**
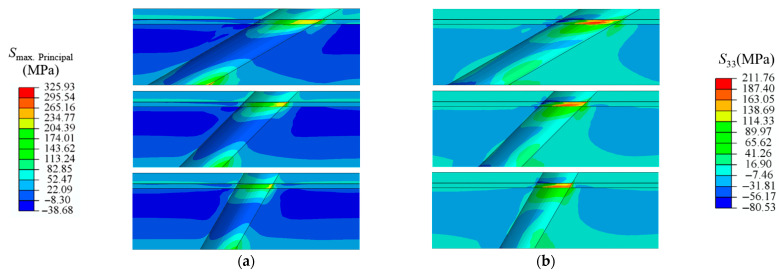

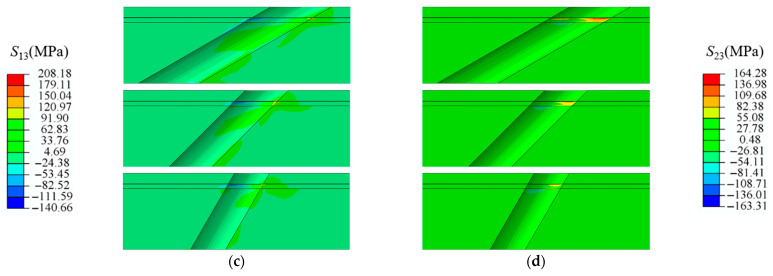
Thermal stress of TBC near film-cooling holes with 30°, 45°, and 60° inclination angles. (**a**), *S*_Max_ (**b**), *S*_33_ (**c**), *S*_13_ (**d**), *S*_23_.

Due to the complex three-dimensional temperature field near the holes and the free edge effects caused by the holes themselves, it is necessary to conduct stress analysis and failure studies in the region around the film-cooling holes under actual working conditions. Figure 8 shows the stress contour maps of the TBC near the profiles of film-cooling holes with angles of 30°, 45°, and 60° under rated conditions. The stresses include maximum principal stress S_max. principal_ (*S*_Max_), normal stress (*S*_33_), and shear stresses (*S*_13_ and *S*_23_). The maximum principal stress (*S*_Max_) refers to the maximum tensile stress at a point in a stress state. *S*_Max_ is one of the main driving forces for crack initiation and propagation. Under the action of *S*_Max_, materials are prone to tensile failure, leading to crack formation and growth. In stress concentration areas (such as hole edges and interfaces), the maximum principal stress often reaches the material’s strength limit, promoting crack initiation. Mode I cracks are primarily driven by tensile stress perpendicular to the crack surface, causing the crack surface to open under tensile stress. Mode II cracks are mainly driven by shear stress parallel to the crack surface, causing relative sliding of the crack surfaces under shear stress. This study selects normal stress *S*_33_ to investigate the induction of Mode I cracks and shear stresses *S*_13_ and *S*_23_ to study the induction of Mode II cracks.

In Figure 8, the inclined hole structure leads to uneven stress distribution, primarily reflected at the hole edges. Under rated conditions, stress is generally concentrated in the BC layer and the TC layer near the film-cooling holes. Larger areas of *S*_Max_ and *S*_23_ also appear downstream of the film-cooling holes at the bottom of the SUB layer [31]. Compared to the upstream of the film-cooling holes, the stress characteristics downstream are more pronounced. This will lead to the downstream region of the film-cooling holes failing first. This is due to the combined effects of the free edge effect of the hole structure and the temperature distribution near the holes. Additionally, it can be concluded that under rated conditions, the bottom of the SUB layer near the film-cooling holes and the junction of the BC and TC layers may fail first. Comparing film-cooling holes with inclination angles of 30°, 45°, and 60°, it is observed that as the inclination angle increases, the stress concentration area near the holes significantly reduces, and the maximum thermal stress also decreases.

**Figure 9:**
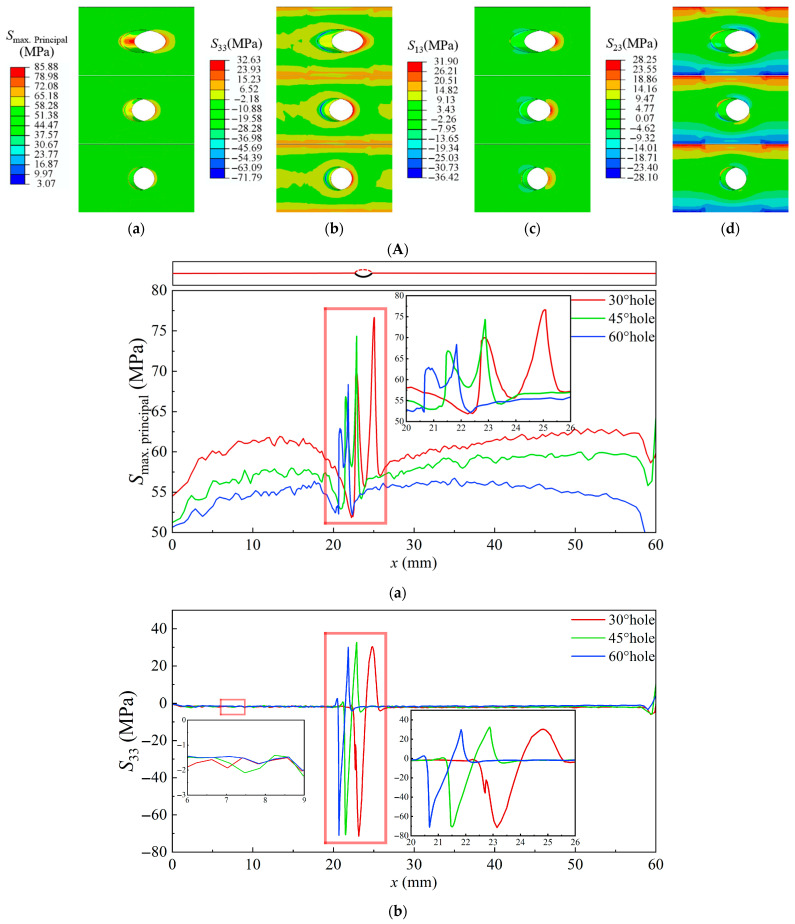

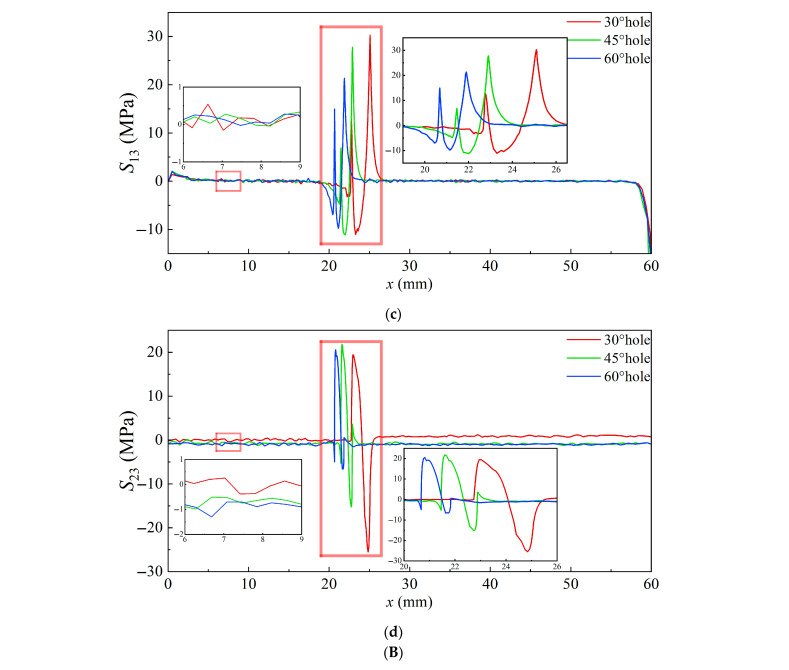
Thermal-stress contour maps and stress distribution diagrams at the TC–TGO interface under rated conditions with 30°, 45°, and 60° inclination angles. (**A**), Thermal-stress contour map at the film-cooling holes (**a**), *S*_Max_ (**b**), *S*_33_ (**c**), *S*_13_ (**d**), *S*_23_. (**B**), Thermal-stress distribution along the central axis and the edge path of film-cooling holes (**a**), *S*_Max_ (**b**), *S*_33_ (**c**), *S*_13_ (**d**), *S*_23_.

Figure 9 illustrates the thermal-stress contour maps around the film-cooling holes at the TC–TGO interface with different inclination angles, as well as the thermal-stress distribution along the central axis and the edge path of the film-cooling holes. At the TC–TGO interface, the maximum *S*_Max_ appears downstream at the hole edge, with significant stress also present in the upstream area, showing a trend of first decreasing and then increasing along the edge path [28]. The normal stress *S*_33_ is compressive upstream of the hole and tensile downstream, transitioning from compressive to tensile along the edge path, with the maximum tensile stress *S*_33_ located downstream. Comparing film-cooling holes with inclination angles of 30°, 45°, and 60°, *S*_Max_ decreases as the inclination angle increases, the influence range of *S*_Max_ also decreases with increasing inclination angle, but the inclination angle has little effect on the normal stress *S*_33_. The maximum shear stress *S*_13_ appears downstream of the hole, and as the inclination angle increases, the stress influence range and maximum stress value decrease [30]. Around the hole, *S*_23_ exhibits a symmetrical opposite distribution, with differences between upstream and downstream areas; downstream stress values are larger, with a wider distribution range, making the left and right ends downstream more prone to inducing Mode II cracks. Upstream of the hole, changes in inclination angle have little effect on *S*_23_, but downstream, the *S*_23_ at a 30° inclination is significantly higher than at 45° and 60° inclinations.

**Figure 10:**
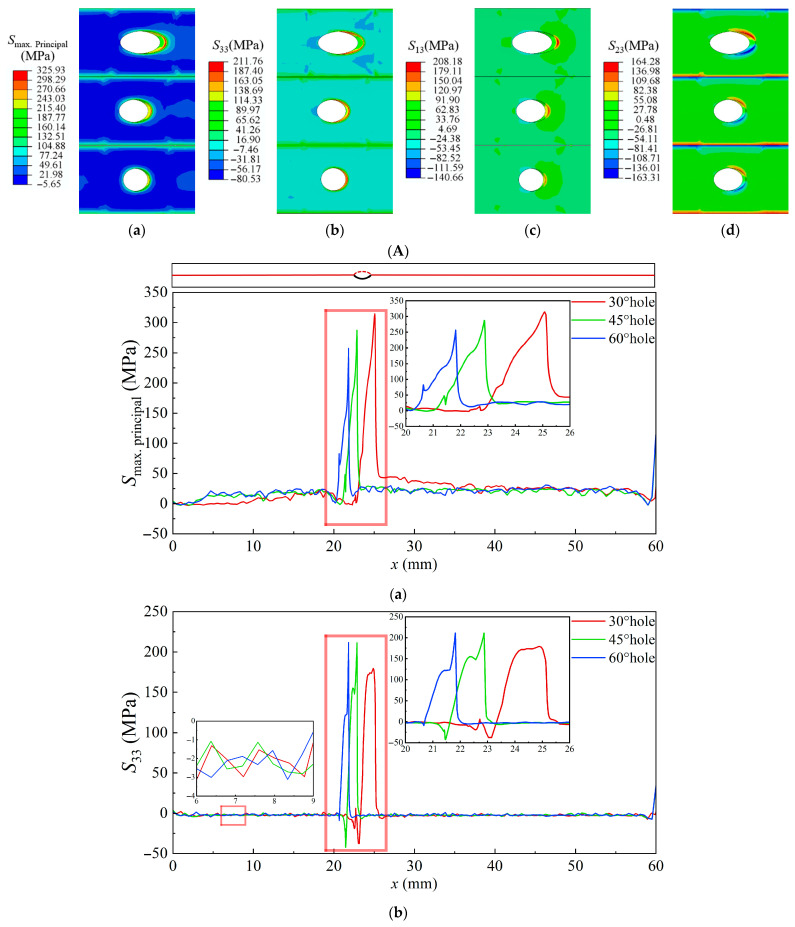

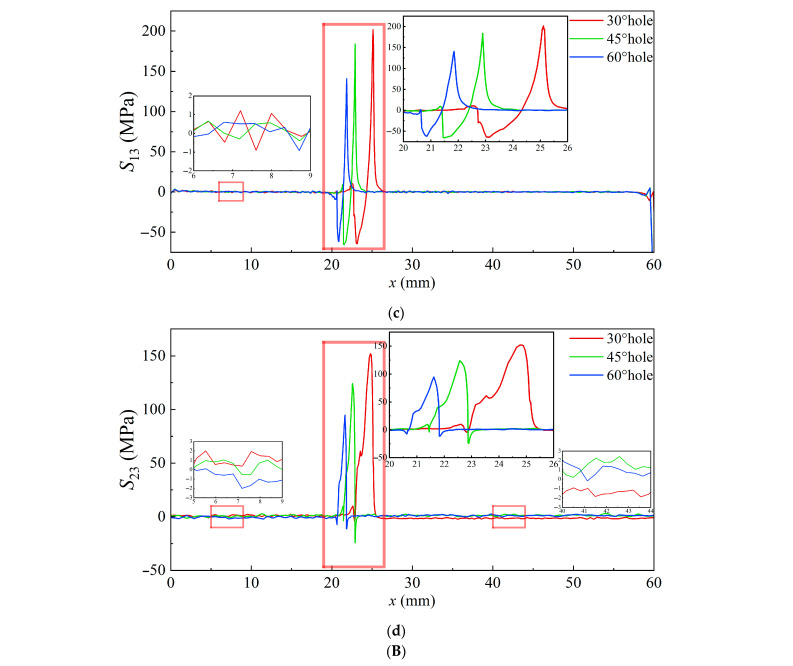
Thermal-stress contour maps and stress distribution diagrams at the BC–TGO interface under rated conditions with 30°, 45°, and 60° inclination angles. (**A**), Thermal-stress contour map at the film-cooling holes (**a**), *S*_Max_ (**b**), *S*_33_ (**c**), *S*_13_ (**d**), *S*_23_. (**B**), Thermal-stress distribution along the central axis and the edge path of film-cooling holes (**a**), *S*_Max_ (**b**), *S*_33_ (**c**), *S*_13_ (**d**), *S*_23_.

The thermal-stress contour maps at the BC–TGO interface and the thermal-stress distribution along the path are shown in Figure 10. Similar to the stress distribution at the TC–TGO interface, stress at the BC–TGO interface is concentrated near the film-cooling holes. *S*_Max_ continuously increases along the edge path of the hole, reaching its maximum at the downstream top of the hole. The normal stress *S*_33_ shows a transition from compressive to tensile along the edge path. The compressive stress upstream of the 60° hole is not obvious, while the maximum compressive stress *S*_33_ upstream of the 30° and 45° holes is 50 MPa greater than that of the 60° hole, better suppressing Mode I crack formation. Downstream near the hole, the tensile stress *S*_33_ for the 45° and 60° holes shows a sharp increase, with maximum *S*_33_ greater than that of the 30° hole, making Mode I cracks more likely to occur downstream of the 45° and 60° holes. The distribution of shear stresses *S*_13_ and *S*_23_ is similar to that at the TC–TGO interface, with greater shear forces in the downstream region of the film-cooling holes, making Mode II cracks more likely, and *S*_13_ and *S*_23_ decrease with increasing inclination angle.

Considering both normal and shear stresses, the stress distribution downstream of the holes is mixed. Under rated conditions, mixed-mode (I + II) cracks may appear downstream of the film-cooling holes at both the TC–TGO and BC–TGO interfaces.

### 3.4. Residual Stress at Room Temperature

The analysis in Section 3.3 was conducted under the rated operating conditions of actual working environments. However, the failure of thermal barrier coatings (TBCs) typically occurs under thermal cycling conditions, especially during the cooling phase, where residual stress significantly influences the initiation and propagation of cracks [38,39]. Additionally, studies [16,35] using simulation and post-mortem analysis of turbine blades in service have shown that the thickening of the thermally grown oxide (TGO) layer is primarily temperature-dependent. The temperature near the film-cooling holes is the lowest, resulting in the TGO layer in these regions being much thinner than in other areas. This indicates that the growth of the TGO layer does not play a decisive role in the spallation of the coating near the film-cooling holes. Therefore, this section focuses on analyzing the residual stress in TBCs after 500 h of creep cooling to room temperature, without considering the growth of the TGO layer.

**Figure 11:**
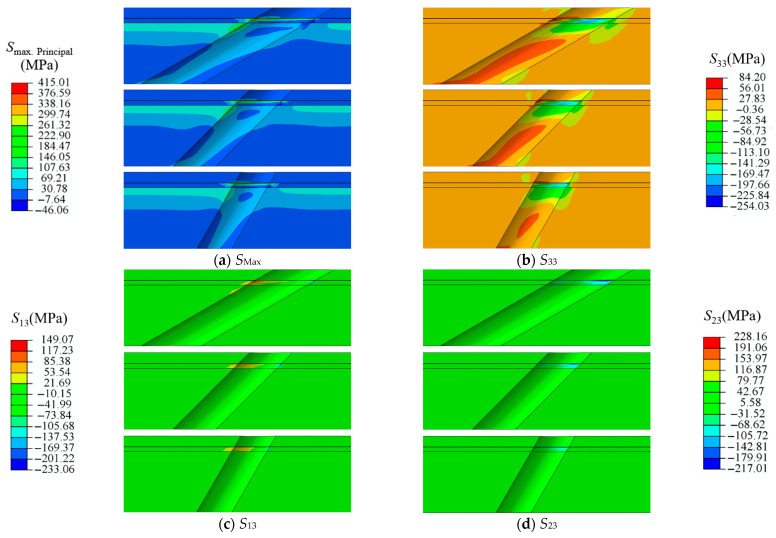
Residual stress of TBC near film-cooling holes with 30°, 45°, and 60° inclination angles. (**a**), *S*_Max_ (**b**), *S*_33_ (**c**), *S*_13_ (**d**), *S*_23_.

Figure 11 shows the residual-stress distribution near the cross-section of film-cooling holes with inclination angles of 30°, 45°, and 60° at room temperature. Compared to thermal stress at high temperatures, residual stress at room temperature poses a greater threat to the TBC material. The distribution of residual stress at room temperature is opposite to that of thermal stress at high temperatures. At room temperature, *S*_Max_ is concentrated at the upstream side of the film-cooling hole at the interface between the TC and the BC, and its value is 90 MPa higher than the *S*_Max_ at high temperatures. The *S*_33_ stress upstream of the film-cooling hole changes from compressive to tensile, while downstream it changes from tensile to compressive. From a numerical perspective, the *S*_33_ stress at room temperature alleviates the initiation of Mode I cracks compared to high temperatures. However, when comparing the shear stresses *S*_13_ and *S*_23_, the cooling process to room temperature makes the initiation of Mode II cracks more likely. Nevertheless, as the inclination angle of the film-cooling hole increases, the stress concentration region near the hole significantly decreases, and the maximum residual stress also reduces accordingly.

**Figure 12:**
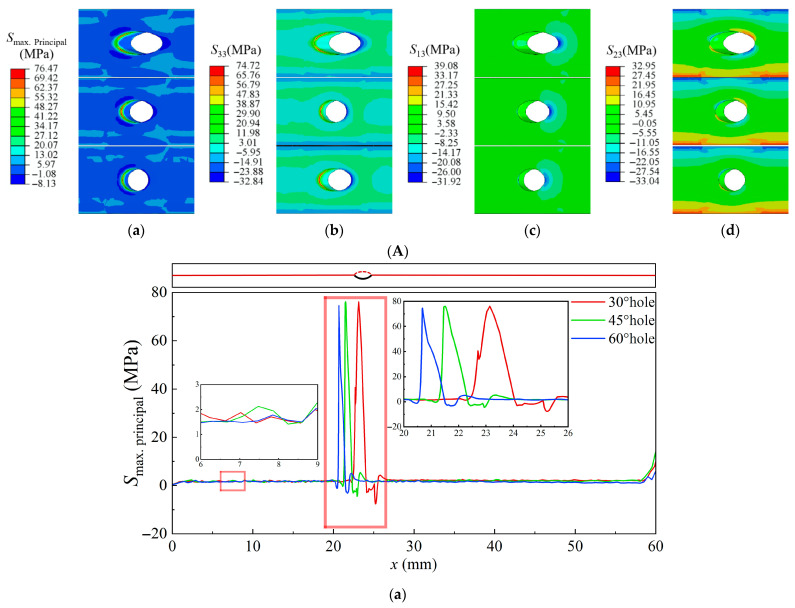

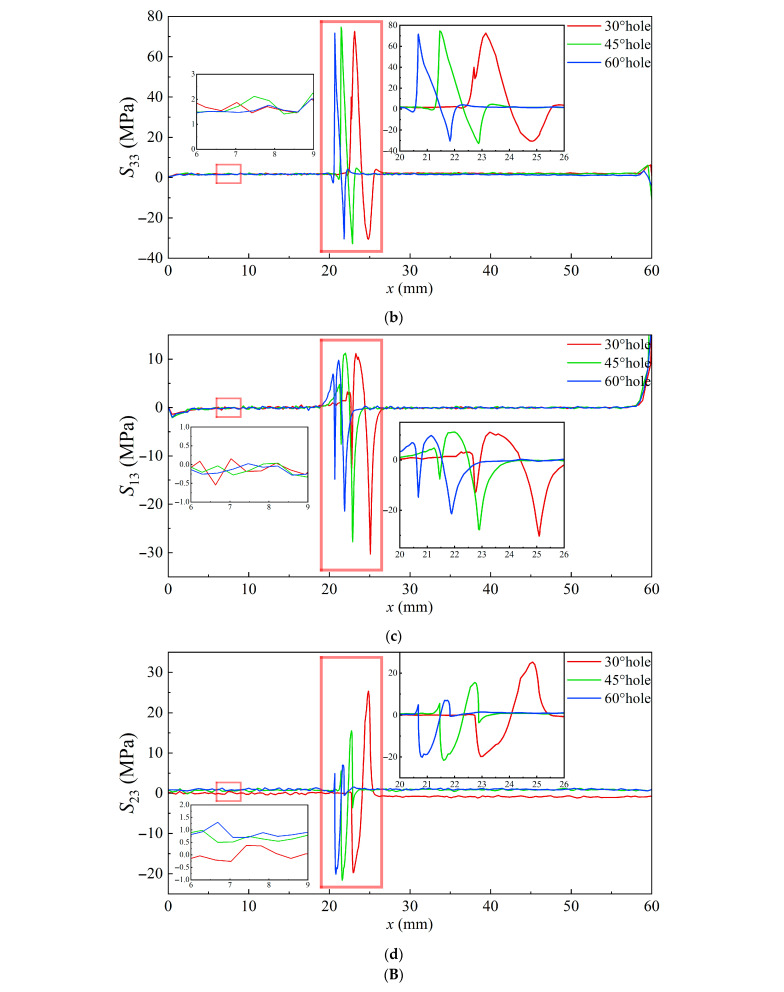
Residual-stress contour maps and stress distribution diagrams at the TC–TGO interface at room temperature for inclination angles of 30°, 45°, and 60°. (**A**), Residual-stress contour map at the film-cooling hole (**a**), *S*_Max_ (**b**), *S*_33_ (**c**), *S*_13_ (**d**), *S*_23_. (**B**), Residual-stress distribution along the central axis and the edge path of film-cooling holes (**a**), *S*_Max_ (**b**), *S*_33_ (**c**), *S*_13_ (**d**), *S*_23_.

After 500 h of creep cooling to room temperature, the residual-stress contour maps around the film-cooling holes at the TC–TGO interface with different inclination angles, as well as the residual-stress distributions along the central axis and the edge path of the film-cooling holes, are shown in Figure 12. Compared to Figure 9, it can be observed that the high-temperature thermal stress and the residual stress after creep at the TC–TGO interface exhibit similar distribution patterns. Compared to high-temperature thermal stress, the influence of the film-cooling hole inclination angle on *S*_Max_ and *S*_33_ is not significant. The region of high *S*_Max_ shifts from the downstream side of the hole to the upstream side. Along the edge path of the film-cooling hole, *S*_33_ transitions from tensile stress to compressive stress, with the maximum tensile stress *S*_33_ located upstream of the hole, which induces the formation of Mode I cracks. For the 30° hole, the maximum tensile stress *S*_33_ after creep increases from 24 MPa under rated conditions to 72 MPa, making Mode I cracks more likely to occur after creep. The shear stresses *S*_13_ and *S*_23_, compared to those under rated conditions, show no change in magnitude around the film-cooling hole but exhibit opposite directions. The maximum shear stress appears downstream of the hole.

**Figure 13:**
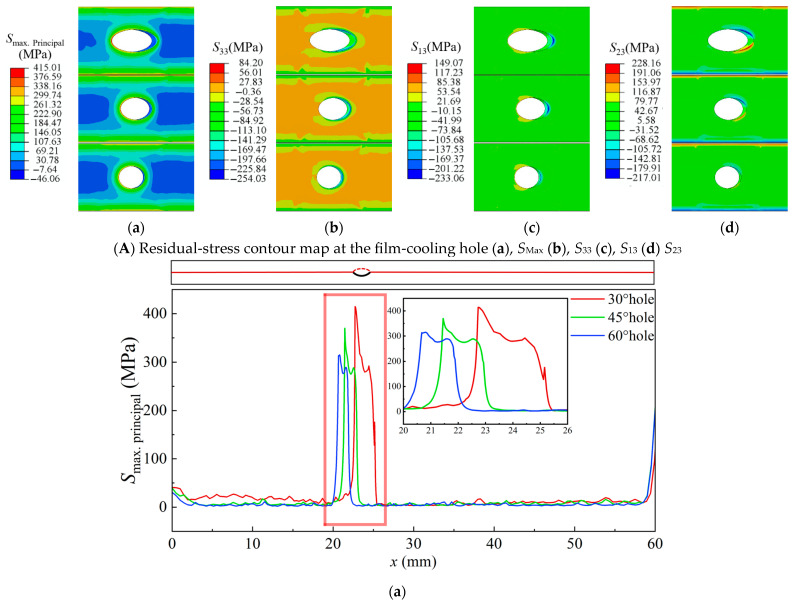

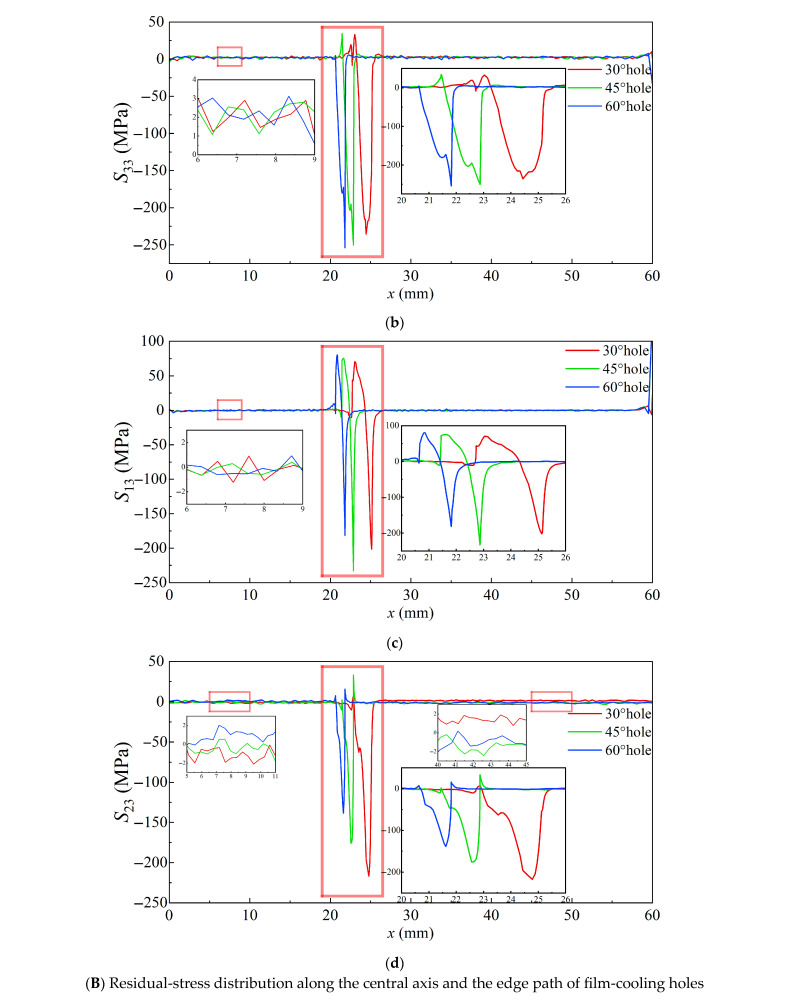
Residual-stress contour maps and stress distribution diagrams at the BC–TGO interface at room temperature for inclination angles of 30°, 45°, and 60°. (**A**), Residual-stress contour map at the film-cooling hole (**a**), *S*_Max_ (**b**), *S*_33_ (**c**), *S*_13_ (**d**), *S*_23_. (**B**), Residual-stress distribution along the central axis and the edge path of film-cooling holes (**a**), *S*_Max_ (**b**), *S*_33_ (**c**), *S*_13_ (**d**), *S*_23_.

The residual-stress contour maps at the BC–TGO interface and the thermal-stress distributions along the path are shown in Figure 13. The residual stress at the BC–TGO interface at room temperature is similar to the high-temperature thermal-stress distribution, but the magnitude of the stress, especially near the hole, is significantly higher. The normal stress *S*_33_ and the shear stresses *S*_13_ and *S*_23_ have opposite directions, and the *S*_Max_ distribution is also reversed. After creep, the high tensile stress downstream of the BC–TGO interface transforms into high compressive stress, which can suppress the formation of Mode I cracks. In the downstream region of the film-cooling hole, the maximum shear stress *S*_13_ for the 45° hole is greater than that for the 30° and 60° holes. After creep, the maximum residual stress around the film-cooling holes with inclination angles of 30°, 45°, and 60° generally increases by 50 MPa, making crack formation during the cooling process more likely. At room temperature, cracks are more prone to occur around the film-cooling holes at the BC–TGO interface compared to high temperatures. At the TC–TGO and BC–TGO interfaces at room temperature, Mode I cracks are more likely to appear upstream of the film-cooling holes, while Mode II cracks are more likely to appear downstream.

Comparing Figure 9, Figure 10, Figure 12 and Figure 13, it can be observed that after prolonged creep relaxation, the TBC is almost in a stress-free state. Therefore, the cooling process from zero stress at high temperatures to room temperature can be considered as the reverse of heating to high temperatures, which explains the similarity in stress distribution before and after creep. However, the residual stress in the BC layer significantly increases, which is due to the plastic accumulation of the BC layer material at high temperatures.

The likelihood of crack formation in 30°, 45°, and 60° holes decreases as the inclination angle increases. From previous analysis, the 30° film-cooling hole offers the best cooling performance. Therefore, when designing the inclination angle of film-cooling holes, it is important to consider not only the cooling performance but also the spallation failure of the TBC. For the model studied in this paper, considering both cooling performance and TBC failure, the 45° film-cooling hole is the optimal choice. Due to the free edge effect of the holes, the optimization of film-cooling hole structures should also comprehensively account for both cooling performance and TBC failure to determine the best design.

## 4. Conclusions

In this study, a numerical model was established using CHT and sequential thermal-stress calculation methods to simulate the temperature field and stress field of the TBC around film-cooling holes under rated operating conditions. Material plasticity and creep were considered to analyze the three-dimensional temperature field, high-temperature thermal stress, and residual stress after 500 h of creep for film-cooling holes with different inclination angles (30°, 45°, and 60°). Additionally, the balance between cooling performance and stress for film-cooling holes at different inclination angles was explored.

The region near the film-cooling holes exhibits a complex three-dimensional temperature field distribution. A sharp temperature gradient is generated in the TBC near the film-cooling holes, and an inverse temperature gradient appears in the TC layer at the film-cooling hole outlet. Outside the film-cooling influence zone, the temperature in other areas of the TBC layers decreases as the inclination angle of the film-cooling holes increases, leading to improved cooling performance.

The TBC around the film-cooling holes exhibits significant stress concentration. At room temperature, Mode I cracks are more likely to occur upstream of the film-cooling hole, while Mode II cracks are more likely to form downstream. Under rated operating conditions, mixed-mode (I + II) cracks are prone to appear downstream of the hole. The coating experiences higher stress at room temperature, indicating that it is more susceptible to spallation failure during the cooling phase.

As the inclination angle of the film-cooling holes increases, the downstream coverage of the cooling film in both the streamwise and lateral directions decreases significantly, leading to a reduction in film-cooling effectiveness. However, the stress concentration in the TBC near the film-cooling holes is alleviated with the increase in inclination angle, reducing the likelihood of crack formation around the holes. Considering both cooling performance and TBC failure, a 45° film-cooling hole is optimal.

The results of this study provide important theoretical support for optimizing the design of film-cooling holes, which helps improve the durability and reliability of coatings. It also offers theoretical backing for optimizing turbine blade design. This has broad engineering application value.

## Figures and Tables

**Table 1:** Material parameters for each layer in the solid domain [29,30,31].

	*T* °C	*k* W/(m·K)	*C* J/(kg·K)	ρ kg/m^3^	*E* GPa	ν	α 10^−6^/°C
TC	25	1.05	483	5650	12.4	0.2	9.68
	800						9.88
	1000						10.34
TGO	25	25.2	857	3978	312	0.27	5.1
	1000						9.8
BC	25	4.3	501	7320	120	0.3	10.3
	400	6.4	592				12.5
	800	10.2	781				14.4
	1000	11.6	764				16.0
SUB	100	11.4	544	8110	139	0.3	12.6
	300	14.9					13.1
	500	18.3					14.0
	700	21.8					14.6
	900	25.2					15.8
	1100	28.7					16.3

**Table 2:** Material plasticity parameters [35].

	*T* (°C)	$\sigma_{y}$ (MPa)	$\epsilon_{y}$
BC	400	1100	0
	400	2500	0.23
	800	300	0
	800	375	0.022
	1000	11	0
	1000	19	0.01
SUB	27	708	0
	27	716	1.998 × 10^−3^
	27	830	0.0402
	600	627	0
	600	636	1.998 × 10^−3^
	600	991	0.0862
	1000	204	0
	1000	205	1.998 × 10^−3^
	1000	355	0.2390

**Table 3:** Material creep parameters [16,36].

	*T* °C	*B* (s^−1^ MPa^−n^)	*m*
TC	1000	1.8 × 10^−8^	1.3
TGO	1000	7.3 × 10^−9^	1
BC	≤600	6.54 × 10^−19^	4.57
	700	2.2 × 10^−12^	2.99
	800	1.84 × 10^−7^	1.55
	≥850	2.15 × 10^−8^	2.45
SUB	800	3.89 × 10^−10^	6.6

## Data Availability

The original contributions presented in this study are included in the article. Further inquiries can be directed to the corresponding author(s).
